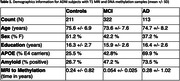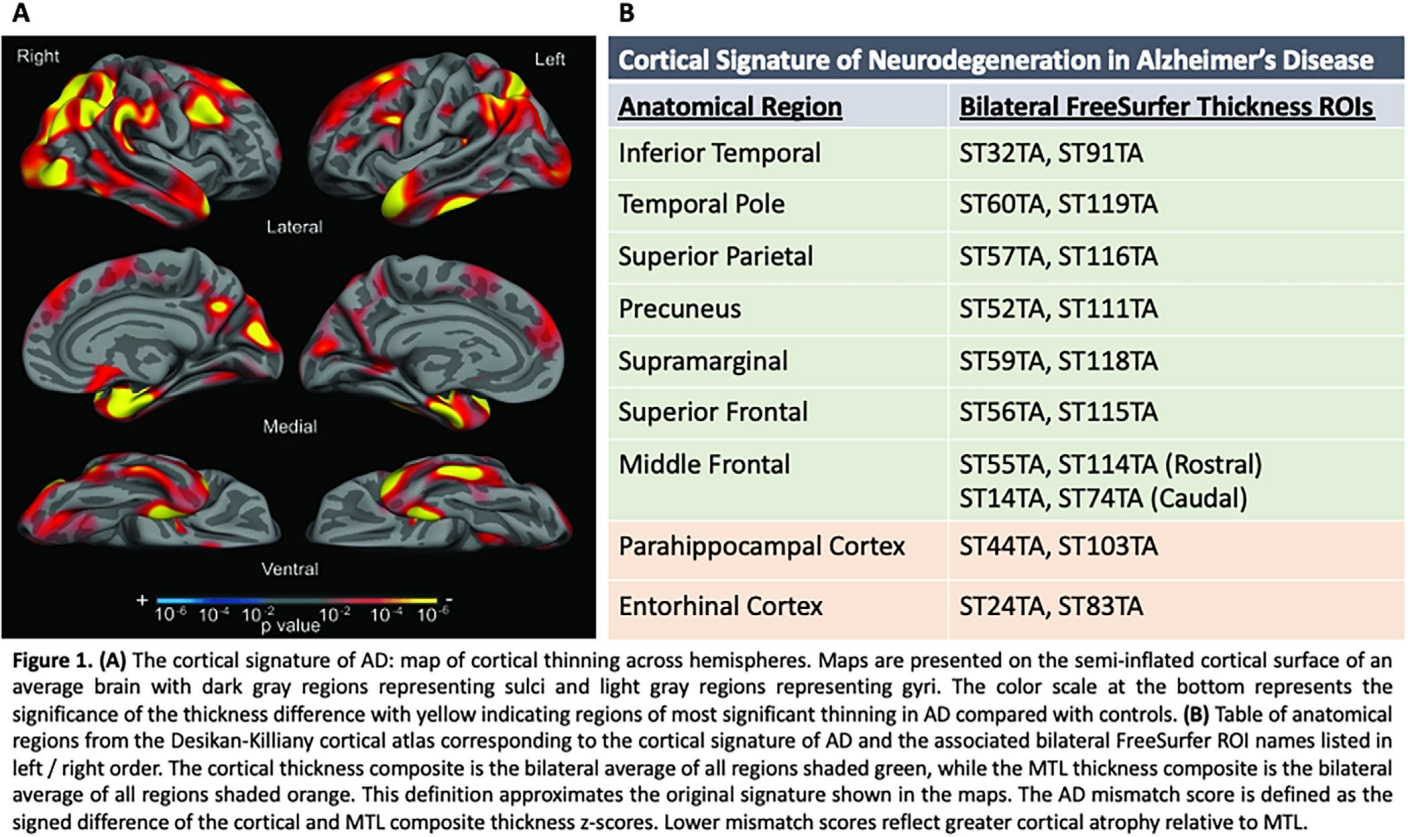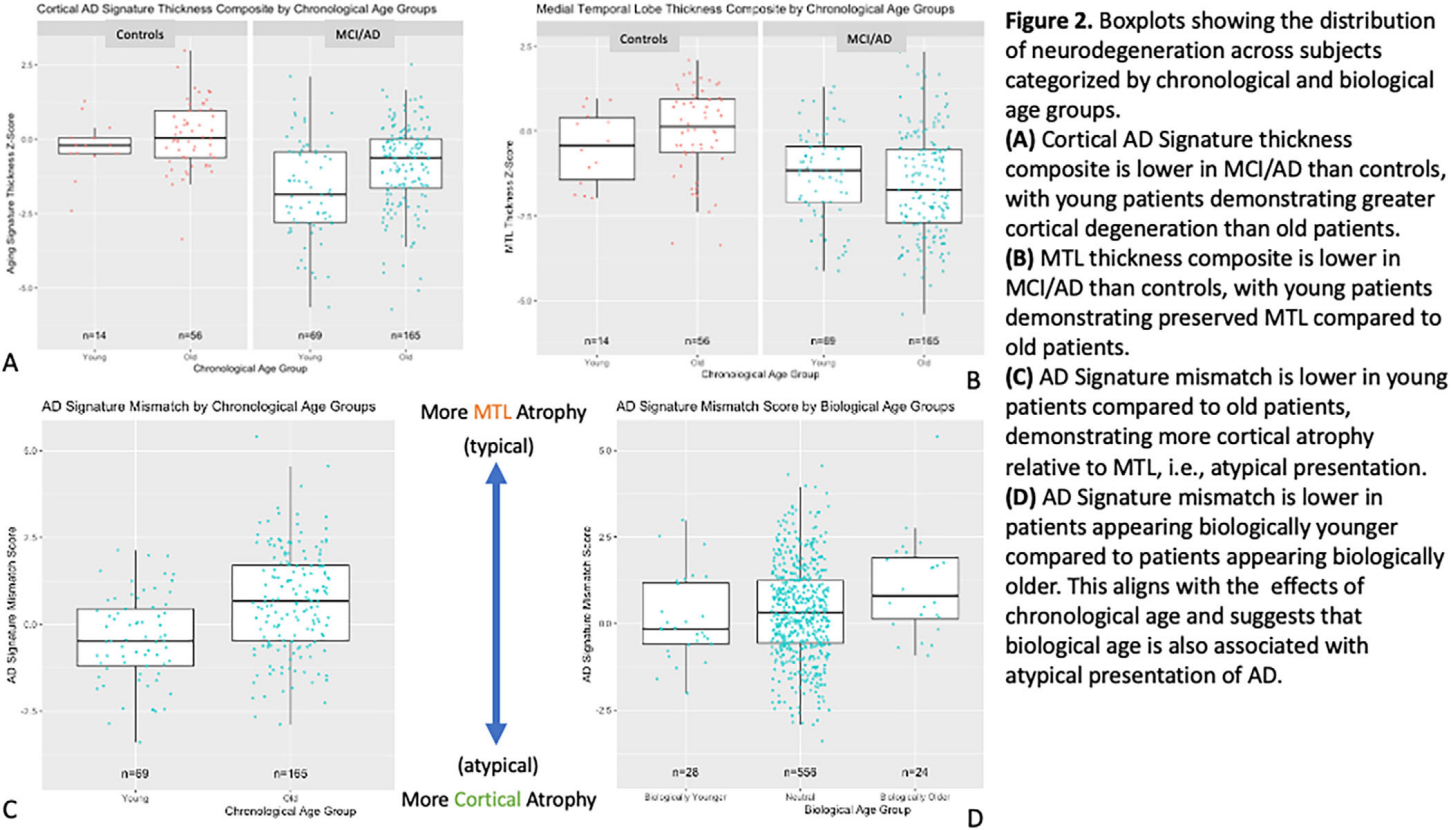# Biological aging is associated with atypical neurodegenerative patterns in Alzheimer's disease

**DOI:** 10.1002/alz.094143

**Published:** 2025-01-09

**Authors:** Lasya P Sreepada, Christopher Brown, Sandhitsu R. Das, Paul A. Yushkevich, David A Wolk, Corey T McMillan

**Affiliations:** ^1^ University of Pennsylvania, Philadelphia, PA USA; ^2^ Penn Alzheimer's Disease Research Center, University of Pennsylvania, Philadelphia, PA USA; ^3^ Penn Frontotemporal Degeneration Center, Department of Neurology, Perelman School of Medicine, University of Pennsylvania, Philadelphia, PA USA

## Abstract

**Background:**

Atypical presentations of Alzheimer’s disease (AD), which demonstrate more cortical involvement relative to medial temporal lobe (MTL), are generally associated with younger age of onset. Age, defined chronologically, is a primary driver of AD pathology and neurodegeneration. However, some young onset cases are typical, amnestic presentations and some older onset cases present more cortical atrophy. We hypothesize that a biological definition of age, defined using epigenetic clock measures, may be even more tightly linked to atypicality than chronological age.

**Method:**

Subjects from ADNI with blood DNA methylation (DNAm) samples and T1 MRI (N=646, 55.7% female, 75.5 +/‐ 7.4 years, Table 1) were selected. Participants were defined as “young” (< 65 years) or “old” (> 80 years) amyloid‐negative controls or amyloid‐positive MCI/AD. DNAm was assayed on Illumina EPIC arrays. Epigenetic age was computed by applying the Shireby cortical epigenetic clock. We then defined epigenetic age acceleration (EAA) by regressing epigenetic age against chronological age and extracting the residual. Subjects were classified as appearing “biologically younger” (EAA < 1 SD below the mean), “neutral” (EAA within 1 SD), or “biologically older” (EAA > 1 SD above the mean). Regional cortical thickness measures were derived from MRI using FreeSurfer 5.1 and harmonized for scanner differences using longitudinal ComBat. We computed composite bilateral thickness z‐scores, adjusted for age and sex relative to controls, in the cortical AD signature anatomical ROIs (Figure 1) and the MTL (entorhinal and parahippocampal cortices). Finally, we defined an AD “mismatch” score as the signed difference of the composite cortical and MTL thickness z‐scores.

**Result:**

Cortical thickness z‐scores were lower in MCI/AD relative to controls. Lower mismatch scores, reflecting greater cortical neurodegeneration relative to MTL, were observed in young‐onset relative to older‐onset MCI/AD. Similarly, mismatch scores were significantly lower in biologically younger cases relative to biologically older cases (t=2.5589; p = 0.01; Figure 2).

**Conclusion:**

Our results support the notion that biological age, as measured by epigenetic modifiers, modulates the degree of atypicality in AD patients, akin to chronological age. These findings suggest that biological aspects of aging may influence the topography of AD‐related pathology and spread.